# The mitogenome mutation repertoire affects progression of Parkinson’s Disease

**DOI:** 10.1590/1678-4685-GMB-2025-0098

**Published:** 2026-02-09

**Authors:** Gustavo Barra Matos, Camille Sena dos Santos, Letícia Cota Cavaleiro de Macêdo, Juliana Paiva dos Santos Diniz, Tatiane Piedade de Sousa, Giovanna Chaves Cavalcante, Caio Santos Silva, Rebecca Lais da Silva Cruz, Dafne Dalledone Moura, Andrea Ribeiro-dos-Santos, Bruno Lopes Santos-Lobato, Gilderlanio Santana de Araújo

**Affiliations:** 1 Universidade Federal do Pará, Instituto de Ciências Biológicas, Laboratório de Genética Humana e Médica, Belém, PA, Brazil.; 2 Universidade Federal do Pará, Instituto de Tecnologia, Belém, PA, Brazil.; 3 Universidade Federal do Pará, Laboratório de Neuropatologia Experimental, Belém, PA, Brazil.

**Keywords:** Mitogenome, transversion, transitions, Parkinson´s disease, Levodopa-induced dyskinesia

## Abstract

Mitochondrial genome variation is a risk factor for Parkinson’s disease, but its role in levodopa-induced dyskinesia remains incompletely understood. This study examines the mitochondrial mutation repertoire as a potential biomarker for levodopa-induced dyskinesia in patients with Parkinson’s disease. We analyzed the mitogenome using next-generation sequencing data from 42 controls and 45 people with Parkinson’s (25 without dyskinesia and 20 with dyskinesia). The *mtDNA-server 2* workflow was applied for variant calling analysis. Transition and transversion rates vary during disease progression, especially in patients without levodopa-induced dyskinesia. Although the occurrence of these mutations does not follow a linear pattern, the frequency of transitions modestly increases with age. Specific coding regions (*CO1, CO2, CO3, ND4, ND5,* and *ND6*) and the regulatory region (*RNR2)* exhibited an enrichment of transitions and transversions in patients without dyskinesia. Additionally, we have upgraded the *mtDNA-network* tool (https://apps.lghm.ufpa.br/mtdna) with an integrated visual component that summarizes the mitochondrial profile in Parkinson’s disease. The study highlights dynamic shifts in the mitochondrial mutation repertoire, with clinical implications for underrepresented populations, underscoring the importance of accounting for genetic characteristics across diverse groups.

## Introduction

Parkinson’s Disease (PD) is a complex neurodegenerative condition primarily characterized by motor symptoms such as tremor, rigidity, and bradykinesia. However, it can also involve a variety of non-motor manifestations. Recent studies have explored the role of mitochondrial DNA (mtDNA) variants in genomic instability contributing to the pathogenesis of P (Dölle et al., 2016; Wu et al., 2018; Epifane-de-Assunção et al., 2024). The context of mtDNA variation generated by next-generation sequencing has been relatively scarce in the scientific literature for PD. Therefore, some studies highlighted the importance of mtDNA variations and their impact on susceptibility and the development of PD (Wu *et al.,* 2018; Tzeng, 2022; Müller-Nedebock et al., 2022; Liu et al., 2023). A recent study showed associations between mtDNA haplogroups and reduced risk of cognitive decline in people with PD, with no effect on motor progression (Liu *et al.,* 2023). One pioneering study investigated somatic variations in the substantia nigra and frontal cortex and reported genetic influences on the mtDNA complex IV/electron transport chain in PD (Coxhead et al., 2016). Also, mutations in the transfer RNA (tRNA) and ATP6 regions are associated with PD in Mexicans (García et al., 2019).

Furthermore, the impact of mtDNA variants on the clinical phenotype of PD remained poorly explored. There is also no data on the association between mitochondrial genome variation and levodopa-induced dyskinesia (LID), a common complication of PD treatment characterized by involuntary, uncontrolled movements, often associated with prolonged levodopa use (Kwon et al., 2022).

Most studies rely on cohorts predominantly of European ancestry, constraining the applicability of their findings to other populations, especially those underrepresented in genomic studies, such as Latin Americans or Native Americans (Schumacher‐Schuh et al., 2022). The lack of mitogenome-wide association studies and susceptibility to PD in Brazilian populations is evident. The Brazilian population is genetically structured, with tri-hybrid proportions of African, European, and Native American ancestries diverging across regions, and populations throughout Latin America exhibit significant genetic diversity (Kehdy et al., 2015). The genetic architecture of admixed populations has been identified as a source of novel population-impactful variants related to PD and other complex diseases (Loesch et al., 2021).

Thus, the present study aims to investigate the mitogenome-wide repertoire of transitions (TSs) and transversions (TVs) in people with PD and LID. To the best of our knowledge, it is the first to report shifts of TSs and TVs in LID, in a population exhibiting mtDNA haplogroups characteristic of Native American ancestry.

## Subjects and Methods

### Study design and participants

We conducted an observational cross-sectional study to characterize the repertoire of TSs and TVs in a comprehensive analysis of the mitochondrial genome of people with PD and control subjects. A total of 87 participants were recruited (45 people with PD and 42 controls) from the Movement Disorders Clinic of Hospital Ophir Loyola in Belém, Brazil. For analyses, participants were divided into three groups: Controls (n = 42), people with PD without LID (NLID, n = 25), and people with PD and LID (LID, n = 20). All participants were examined by the same movement disorder specialist (B.L.S-L.), and clinical and epidemiological information was collected. People with PD and controls were included, matched for sex and age, with a maximum difference of 4 years. We excluded participants with acute or chronic infectious diseases, severe systemic conditions, autoimmune disorders, or other neurological diseases.

All individuals with PD met the clinical diagnostic criteria of the UK Parkinson’s Disease Society Brain Bank. Control subjects were recruited from a voluntary cohort. The study was approved by the Ophir Loyola Hospital Ethics Committee (number 3.002.664) and all participants provided written informed consent.

### Sample collection, DNA extraction, amplification, and sequencing

Peripheral blood samples were obtained through intravenous puncture, stored at -20ºC after collection in EDTA-containing tubes, and subsequently used for DNA extraction. The extraction process employed the phenol-chloroform protocol with specific adjustments, along with the KingFisher Nucleic Acid Extractor. DNA quantification was performed using a Nanodrop spectrophotometer, and the samples were diluted to 20 ng/µL. 

The mitogenome was then amplified by polymerase chain reaction using 33 pairs of carefully designed primers, as described by Cavalcante et al. (2019). Library preparation for mitochondrial genome sequencing used the Illumina Nextera XT DNA kit (Illumina Inc., Chicago, IL, USA) according to the manufacturer’s instructions. DNA quality was verified with the high-sensitivity ScreenTape D1000 and in the Agilent 2200 TapeStation system. The actual sequencing was performed on the Illumina MiSeq system using the MiSeq Reagent Kit v3 (600 cycles).

### Bioinformatics analysis

**Figure 1 -:**
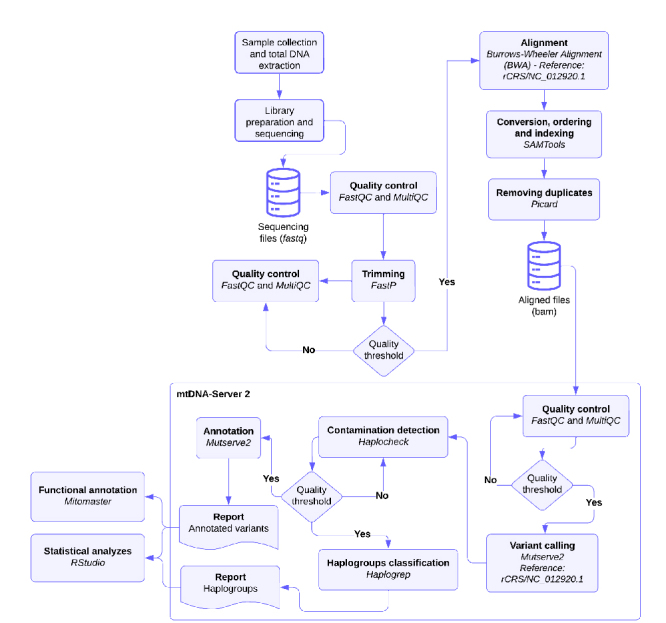
Flowchart for processing mtDNA next-generation sequencing (NGS) data to investigate the occurrence of transitions and transversions.

To assess sequencing quality, we used FastQC (v0.12.1) (Andrews, 2010) and MultiQC (v.1.19) (Ewels et al., 2016) before and after processing the sequencing files. Pre-processing of mtDNA sequencing involved removing low-quality bases (Phred Score: <Q20), sequencing adapters, and reads with a length less than 36 nucleotides using FastP (v0.23.4) (Chen, 2023). After treatment and quality assessment, the files were aligned with the mtDNA reference sequence - Revised Cambridge Reference Sequence (rCRS) - using the Burrows-Wheeler alignment tool (v0.7) (Li and Durbin, 2009). Subsequent processes of mapping, sequence sorting, and duplicate read removal were performed using SAMTools (v.1.15.1) (Danecek et al., 2021) and Picard (v2.27.5) (Broad Institute, 2019), respectively. SNP calling, contamination detection, and haplogroup classification were carried out using the mtDNA-Server 2 workflow (Weissensteiner et al., 2024), which is specific to human mitochondrial variant analysis (Figure 1).

A tolerable limit of 10% contamination was adopted to avoid false positives, minimize the presence of exogenous DNA, and preserve the reliability of haplogroup determination (Weissensteiner et al., 2021). Samples that exceeded this limit were excluded from the study. The set of SNPs was then classified as TSs and TVs.

In addition, read depth and heteroplasmy were used as filters for TSs and TVs. Our analysis considered the overall average depth of variants, as well as heteroplasmy levels ranging from >0.05 to <0.95 to exclude homoplasmic variants.

The sequencing data are deposited in the European Nucleotide Archive (ENA) under accession code PRJEB74357. Raw data can be downloaded at https://apps.lghm.ufpa.br/mtdna.

### Statistical analysis

The coefficient of determination between participants’ ages and TS rates was obtained by fitting a Generalized Additive Model (GAM). The GAM is a statistical technique that allows modeling nonlinear relationships. GAM is useful when relationships between variables cannot be adequately modeled by traditional linear models (Pearson or Spearman). GAM adds smooth functions of each independent variable to the model, allowing for the capture of complex patterns in the data (Rigby and Stasinopoulos, 2005). We employed cubic spline smoothing on the TS data, ensuring that the smoothing process remains linear at the endpoints. This approach helps to mitigate issues related to excessive oscillation or instability at the extremes of the distribution. GAM was not fitted to TV rates, which follow a zero-inflated distribution.

## Results

### Clinical characteristics of participants

Among the groups, PD onset age was lower in LID individuals than in NLID individuals (p = 0.005). Tremor as the first motor symptom (p = 0.023) and the tremor-dominant motor phenotype (p = 0.043) at evaluation were more prevalent in individuals with NLID. L-DOPA dose (p = 0.0003) and L-DOPA therapy duration (p = 0.0006) were higher in individuals with LID. Control individuals had a higher frequency of pesticide exposure (p = 0.011) (Table S1).

### Depth of coverage and mitochondrial haplogroups

The depth of sequencing coverage was analyzed. The average depth coverage before application of the heteroplasmy and coverage filters was 913x, with an interquartile range of 616x to 1416x (Figure S1A). After applying the filters, a median of 965x was observed, with an interquartile range of 696x to 1438x (Figure S1B). In addition, the protein-coding regions (*ND5, CO3, ND4,* and *ATP6*) showed greater depth of coverage.

We conducted mitochondrial haplogroup classification, which showed genetic stratification (typical in admixed populations). We observed a higher prevalence of Native American mitochondrial ancestry, as illustrated in Figure S2A. Specifically, haplogroup C was the most frequent among all groups (Figure S2B). The distribution of haplogroups varied across the studied groups (Control, LID, and NLID), with haplogroups A, B, and C being the most prevalent among patients with Parkinson’s disease. In contrast, haplogroups of African (L0, L1, L2, L3) and Eurasian origin (H, J, T, U) were less represented. This distribution aligns with the expected mitochondrial ancestry for populations in the Amazon region, which is predominantly Native American.

### Transitions and transversions are high in people without levodopa-induced dyskinesia

**Figure 2 -:**
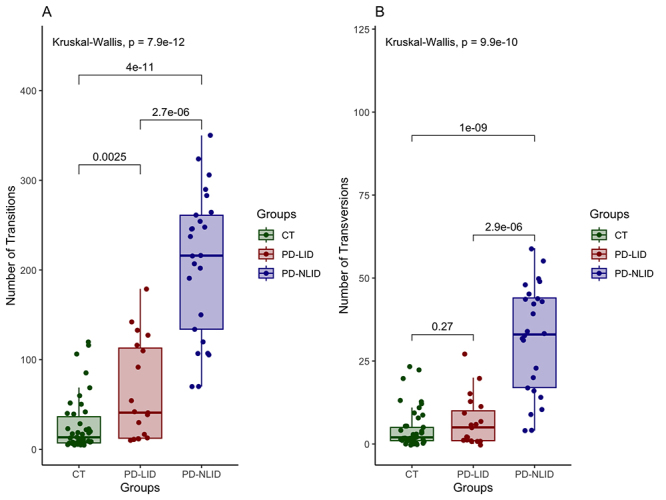
Counts of transitions and transversions across groups. **(A)** Pairwise comparisons and statistical differences in rates of transitions across groups. **(B)** Pairwise comparisons and statistical differences in rates of transversions across groups. Abbreviations: CT: Control group; PD-NLID: People with Parkinson’s disease without levodopa-induced dyskinesia; PD-LID: People with Parkinson’s disease with levodopa-induced dyskinesia.

Statistical differences were evident in the distribution of TSs across the groups (*P* = 7.9e-12). Considering the progress of the disease, we noted a fluctuation in the number of TSs being higher in the PD-NLID group than the severe form, PD-LID group (Figure 2A). Regarding TV comparisons, differences among the groups (*P* = 9.9e-10) were observed. Pairwise comparisons revealed statistically significant differences between controls and PD-NLID groups (*P* = 4e-11) and between PD-NLID and PD-LID groups (*P =* 2.7e-06). Moreover, a similar pattern of TV counts in controls and the PD-LID group was observed (Figure 2B). In addition, we compared the counts of TSs (*P* = 0.041) and TVs (*P* = 0.02) by sex, and a difference between the sexes was noted in the PD-NLID group (Figure S3).

Fisher’s exact test was performed for the number of TSs and TVs in the mtDNA complexes. In Complex IV, there is a statistically significant association in the frequency of VTs in relation to TSs between the PD group and the controls (*OR* = 4.79, *P* = 0.012) and in Complex I (*OR* = 0.74, *P* = 0.018) (Table S2).

**Figure 3 -:**
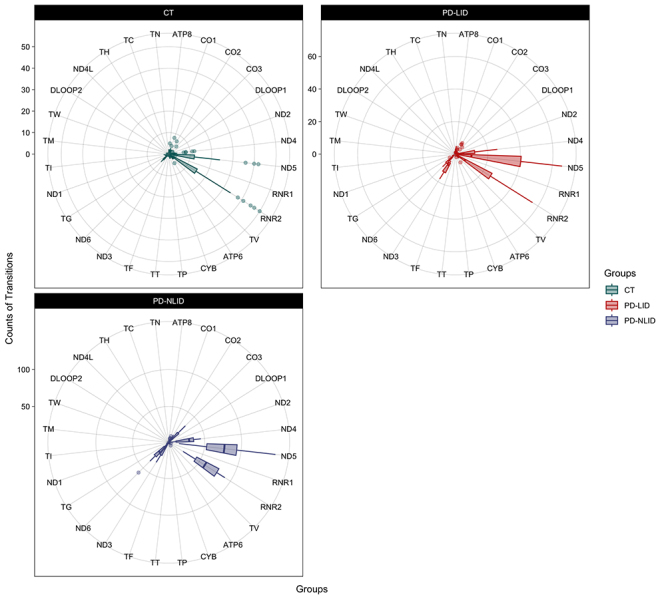
Distribution of TS occurrences in mitochondrial genes. Abbreviations: CT: Control group; PD-NLID: People with Parkinson’s disease without levodopa-induced dyskinesia; PD-LID: People with Parkinson’s disease with levodopa-induced dyskinesia.

We quantified the TS and TV indices by mtDNA region. A greater presence of TSs was noted in the *ND5* and *RNR2* genes (Figure 3). However, there was a significant occurrence of TSs only in the *CO1* (*P* = 0.0091), *CO2* (*P* = 0.027), *CO3* (*P* = 3.4e-09), *ND4* (*P* = 1.8e-09), *ND5* (*P* = 7.5e-11), *ND6* (*P* = 0.0033), and *RNR2* (*P* = 6.7e-10) genes (Figure S4). These regions showed statistically significant differences among the three groups. Other pairwise comparisons between the groups are shown in Figure S4. 

**Figure 4 -:**
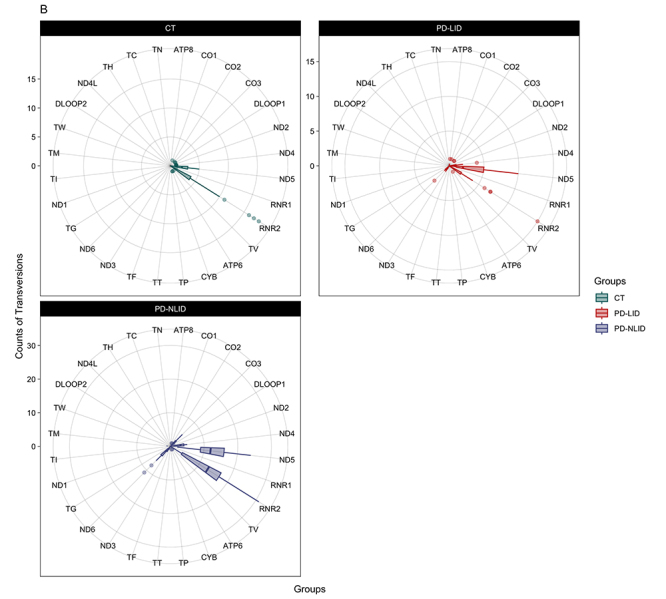
Distribution of TV occurrences in mitochondrial genes. Abbreviations: CT: Control group; PD-NLID: People with Parkinson’s disease without levodopa-induced dyskinesia; PD-LID: People with Parkinson’s disease with levodopa-induced dyskinesia.

Regarding the TV rates, occurrences were in the same regions as TVs (ND5 and RNR2) (Figure 4). However, five genes showed statistically significant differences between all groups: CO3 (P = 9e-08), ND4 (P = 3.4e-09), ND5 (P = 1.4e-10), ND6 (P = 0.0026), and RNR2 (P = 8.8e-07) (Figure S5). Other pairwise comparisons between the groups are shown in Figure S5.

### Age reflects changes in transitions and transversions in Parkinson’s disease

**Figure 5 -:**
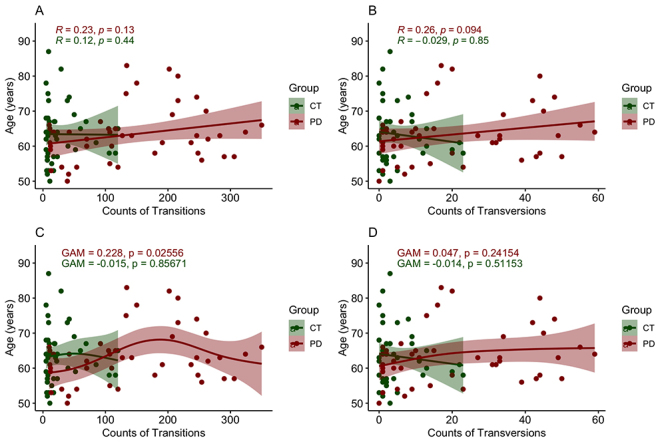
Correlations between counts of transitions and transversions and age in people with Parkinson’s Disease and control individuals. **(A)** Correlations between counts of TSs and age in people with PD and controls; **(B)** Correlations between counts of TVs and age in people with PD and controls; **(C)** GAM correlation of TSs and age in people with PD and controls; **(D)** GAM correlation of TVs and age in people with PD and controls. Abbreviations: CT: control group; PD: people with Parkinson’s disease.

We investigated the statistical relationship between TSs and TVs with age (Figure 5). No correlation was found between age and TSs (Spearman’s correlation *R* ^*2*^ = 0.23, *P* = 0.13) and TVs (Spearman’s correlation *R* ^*2*^ = 0.26, *P* = 0.094) in people with PD (Figure 5A and Figure 5B). In the control group, there was no correlation between age and TSs (Spearman’s correlation *R* ^*2*^ = 0.12, *P* = 0.44) and TVs (Spearman’s correlation *R* ^*2*^ = -0.029, *P* = 0.85) (Figure 5A and Figure 5B).

Following a data distribution analysis, we observed that the relationship between TSs, TVs, and age is not linear. Due to this non-linear behavior, we employed GAM models to assess the determination coefficients for each pair. With this approach, we found that there was no correlation between TSs and age in control (GAM = -0.015, *P* = 0.85671) (Figure 5C). However, in individuals with PD, a weak but statistically significant positive correlation between TSs and age was observed (GAM = 0.228, *P* = 0.02556), suggesting that TSs tend to increase with age in PD (Figure 5C). Regarding TVs, there were no significant correlations between CT and PD (Figure 5D).

### Mitochondrial genetic comorbidity in people with Parkinson’s Disease

By integrating the MITOMAP database data, we found that people with PD harbor intriguing connections between other complex phenotypes and disease-causing mutations. The variant m.13511 (A->T), a TVS in the *ND5* region, that shows 100% conservation and was identified in ten people with PD, is also associated with Leigh-like syndrome. In turn, the variant m.1608 (G>A), a TVs in region V with 72% conservation was detected in four patients people with PD, showing associations with both Leigh Syndrome and Parkinsonism with dystonia (Finsterer, 2024; Saluja et al., 2024).

Another relevant mutation is the variant m.9861 (T>C), a TS in the CO3 region, found in nine individuals with PD. This mutation, with 42% conservation, showed inverse heteroplasmy levels, highlighting its complexity in the context of PD. It is noteworthy that this same mutation is associated with Alzheimer’s disease (Hamblet et al., 2006).

Furthermore, one individual with PD presented the mutation m.15603 (T>C), a TS in *ND5* with 13% conservation, associated with Leber’s hereditary optic neuropathy (LHON). Interestingly, six of these people with PD also carried another mutation, m.7868 (C>T), a transition in the *CO2* region. This mutation is likely to have synergistic effects in the context of LHON. These findings underscore the complexity of relationships between mtDNA genetic variants and other neurodegenerative phenotypes observed in people with PD, calling for an understanding of the molecular implications of these mutations in disease development through a systemic approach (Yang et al., 2009).

### mtDNA-network upgrade

Previously, we developed a graph-based visualization tool designed to analyze cancer-related data (Cavalcante et al., 2022), which has since been enhanced to investigate the relationships between mitochondrial DNA (mtDNA) variants and other complex diseases, including neurodegenerative and infectious diseases. The mtDNA-network tool identifies shared mutations among control, LID, and NLID groups, provides interactive access to raw data for further exploration, and can be visualized at https://apps.lghm.ufpa.br/mtdna/.

## Discussion

Initially, our study characterized the repertoire of TSs and TVs in the mtDNA of controls and individuals with PD. Following the biological pattern, in all groups, the TS index significantly exceeded that of TVs, aligning with the suggested and identified pattern in human mtDNA, where TSs are approximately 15x more frequent than TVs (Belle et al., 2005). People with PD exhibited higher rates of TSs and TVs than controls, suggesting that changes in the occurrence patterns of TSs and TVs are associated with different phases of PD. A previous study examining alterations in the substantia nigra of individuals with PD did not reveal significant differences in TVs between individuals with PD and controls (Dölle et al., 2016). However, the occurrences of TVs in individuals with PD in our study suggest an active involvement of oxidative stress in inducing changes in mtDNA (Caliri et al., 2020). This may be due to methodological differences or specific characteristics of the populations studied, such as ancestry. While the study by Dölle *et al.* (2016) investigated the mtDNA of European individuals, our cohort consisted predominantly of individuals with Native American haplogroups from the northern region of Brazil. Mitochondrial haplogroups exhibit variations in genetic mutation patterns from an evolutionary perspective, and the genetic composition of populations can influence these patterns (Gojobori, 2021; Malyarchuk, 2023). It is known that Native American haplogroups, predominant in a significant portion of our cohort, carry more genetic alterations in mtDNA compared to European haplogroups (Lee and Merriwether, 2015).

LID typically emerges after nearly five years of levodopa therapy, associated with motor dysfunction (Aquino and Fox, 2015). Therefore, LID is often considered a clinical marker of an advanced stage of PD. Our study revealed that the LID group exhibited lower rates of mutations in mtDNA than the NLID group. This change in the mutation spectrum may be explained by inflammatory mechanisms related to PD (Pisanu et al., 2018; Santos-Lobato et al., 2022). 

Additionally, the frequency of G-A transitions is higher in non-neurodegenerative conditions compared to neurodegenerative diseases, suggesting that specific mutational patterns may be indicative of pathological processes (Shen and Ji, 2015). Furthermore, the susceptibility of guanine to oxidative damage results in a higher occurrence of G-T to G-C transversions in neurodegenerative contexts, which may contribute to the pathophysiology of diseases like Parkinson’s (Shen and Ji, 2015).

It is believed that LID results from fluctuations in dopamine levels in the brain due to pulsatile stimulation from levodopa therapy (Hansen et al., 2022). Intense stimulation can trigger inflammatory responses, increasing oxidative stress and generating reactive oxygen species (ROS) that damage mtDNA (Blesa et al., 2015; Chakrabarti and Bisaglia, 2023), especially in the early stages of levodopa therapy in PD (NLID group). After a long period of levodopa treatment, the intense damage caused to mtDNA during the initial stages of treatment may lead to mitochondrial degradation and, consequently, to a reduction in the number of copies and different patterns of expression of the mitochondrial genome (Lowes et al., 2020; Souza et al., 2024). This could explain the reduced rates of mtDNA mutations in individuals with LID.

We observed a preferential occurrence of TSs and TVs in protein-coding regions of mtDNA, particularly within Complex I and Complex IV. Dysfunctions in Complex I are linked with production of ROS, which in turn leads to damage to essential cellular components. Furthermore, Complex I dysfunction can compromise mitochondrial integrity and activate apoptosis pathways, contributing to neurodegeneration (Li et al., 2021). Complex IV is responsible for the electron transport chain, which relies on the reduction of oxygen to water. Dysfunction of Complex IV can also affect ATP production, compromising the metabolic function of neuronal cells. 

The repertoire of TS changes in mtDNA is a phenomenon that occurs with aging and is subject to a process known as clonal expansion (Insalata et al., 2021). Our study highlighted that TSs in people with PD increased with age compared to control individuals. Clonal expansion is particularly significant for understanding phenotypic effects, (Lawless et al., 2020). For instance, clonal expansion may increase in the NLID group and decrease in the LID due to consecutive damage to mtDNA. This hypothesis suggests that mtDNA mutations and clonal expansion may play a pivotal role in the pathogenesis of dyskinesia in Parkinson’s disease and could potentially serve as a biomarker for differentiating between NLID and LID groups and guiding treatment strategies.

One limitation of our study is the recruitment of participants with LID, which is challenging and often requires extended time and resources. While the final sample size of the NLID and LID groups in this study is smaller than anticipated, it reflects the inherent challenges of conducting a longitudinal genetic study for PD.

## Conclusions 

In conclusion, this research significantly improves our understanding of the mitogenome-wide mutation repertoire in the progression of PD and its implications for underrepresented populations, also underscoring the importance of considering the specific genetic characteristics of mixed populations. This study highlights the importance of investigating the mtDNA mutation repertoire to adapt medical care and risk prediction models to the particular genetic contexts within diverse populations. Despite the modest sample size, we acknowledge studies with larger sample sizes and experimental validation. Furthermore, we identified potential biomarkers, shedding light on the complex interplay between genetic variations and the pathophysiology of neurodegeneration and motor phenotypes in LID.

## Supplementary material

The following online material is available for this article:

Table S1 Demographical and clinical characteristics of patients and the control group

Table S2 Number of transitions and transversions and their ratio in the mitochondrial complexes of mtDNA in people with Parkinson’s disease and controls

Figure S1 Distribution of the general depth of coverage

Figure S2 Classification of ancestry and mitochondrial haplogroups

Figure S3 Counts of transitions and transversions by sex across groups

Figure S4 Comparison of transition counts reveals statistical differences in seven mitochondrial genes (*CO1*, *CO2*, *CO3*, *ND4*, *ND5*, *ND6*, and *RNR2*) between the groups

Figure S5 Pairwise comparison of distribution of TVs highlights statistical differences in five mitochondrial genes (*CO3*, *ND4*, *ND5*, *ND6*, and *MT-RNR2*) across groups

## Data Availability

 The sequencing data are deposited in the European Nucleotide Archive (ENA) under accession code PRJEB74357. Raw data can be downloaded at https://apps.lghm.ufpa.br/mtdna.
