# Supplementary material for: The mitogenome mutation repertoire affects progression of Parkinson’s Disease
Source: Genet Mol Biol. 2026 Feb 9;49(Suppl 4):e20250098. doi: 10.1590/1678-4685-GMB-2025-0098 (PMC12965417; doi:10.1590/1678-4685-GMB-2025-0098)
Supplement: Table S1 [file 1415-4757-GMB-49-s4-e20250098-s1.pdf]

Genetics and Molecular Biology
https://doi.org/10.1590/1678-4685-GMB-2025-0098

Supplementary Material to "The mitogenome mutation repertoire affects progression of Parkinson's Disease"

Table S1. Demographical and clinical characteristics of patients and the control group.

Characteristics	Controls (*n* = 42)	NLID (*n* = 25)	LID (*n* = 20)	*P- value*
Sex (Male/Female)	22 (52.4) / 20 (47.6)	17 (68.0) / 8 (32.0)	14 (70.0) / 6 (30.0)	0,321[2]
Age at evaluation[1]	63 (57.2-67)	63(60-70)	61(57-64.2)	0.265[3]
Age at onset[1]	-	57 (53-60)	52.5 (41-57.2)	0.005[5]
Duration of PD (years)[1]	-	5 (5-10)	9 (6-12)	0.084[4]
Duration of LID (Years)[1]	-		2 (1-3.25)	-
Family history of PD (%)				
Yes	-	5 (20.0)	2 (10.0)	
No	-	17 (68.0)	16 (80.0)	0.704[2]
Possibly	-	3 (12.0)	2 (10.0)	
First motor symptom (%)				
Tremor	-	21 (84.0)	10 (50.0)	0.023[2]
Others	-	4 (16.0)	10 (50.0)	
Motor phenotype (%)				
Tremor dominant	-	19 (76.0)	9 (45.0)	
Postural instability with gait disorder	-	5 (20.0)	5 (25.0)	0.043[2]
Indeterminate	-	1 (4.0)	6 (30.0)	
Hoehn & Yahr stage (%)				
1-2	-	22 (88.0)	16 (80.0)	0.682[2]
3-4	-	3 (12.0)	4 (20.0)	
MDS-UPDRS Part 3 (%)				
<35	-	12 (48.0)	15 (75.0)	0.077[2]
>35	-	13 (52.0)	5 (25.0)	
L-DOPA therapy duration (years)[1]	-	4 (3-5)	6 (5-10.2)	0.0006[4]
L-DOPA dose (mg/day)[1]	-	425 (300-681)	862.5 (712.5-1440)	0.0003[4]
Pesticide exposure				
Yes	31 (73.8)	22 (88.0)	15 (75.0)	0.415[2]
No	11 (26.2)	3 (12.0)	5 (25.0)	
Time of pesticide exposure (years)[1]	10 (0-20)	9(2.25-33.8)	15 (0.12-25)	0.473[3]
Frequency of pesticide exposure (days/year)				
No exposure	11 (26.2)	3 (12.0)	5 (25.0)	
1-10	13 (30.9)	2 (8.0)	2 (10.0)	
11-30	7 (16.7)	2 (8.0)	1 (5.0)	0.011[2]
>30	11 (26.2)	17 (68.0)	12 (60.0)	

[1]Values are shown as median (interquartile range); [2]*P-value* obtained by Fisher's Exact test; [3]*P-value* obtained by Kruskal-Wallis test; [4]*P-value* obtained by Mann-Whitney U test; [5]*P-value* obtained by Student's t-test; PD: People with Parkinson's disease; NLID: People with Parkinson's disease without levodopa-induced dyskinesia; LID: People with Parkinson's disease and levodopa-induced dyskinesia.
