# Supplementary material for: The mitogenome mutation repertoire affects progression of Parkinson’s Disease
Source: Genet Mol Biol. 2026 Feb 9;49(Suppl 4):e20250098. doi: 10.1590/1678-4685-GMB-2025-0098 (PMC12965417; doi:10.1590/1678-4685-GMB-2025-0098)
Supplement: Table S2 [file 1415-4757-GMB-49-s4-e20250098-s2.pdf]

Genetics and Molecular Biology
https://doi.org/10.1590/1678-4685-GMB-2025-0098

Supplementary Material to "The mitogenome mutation repertoire affects progression of Parkinson's Disease"

Table S2. Number of transitions and transversions and their ratio in the mitochondrial complexes of mtDNA in people with Parkinson's disease and controls.

Complexes	CT (n = 42)		PD (n = 45)		OR	*P- value*[1]	95% CI
	Transitions	Transversions	Transitions	Transversions			
Complex I	480	90	3806	529	0.740	0.018	0.58-0.94
Complex III	13	2	31	1	0.230	0.235	0.00-3.07
Complex IV	145	2	507	36	4.79	0.012	1.34-32.06
Complex V	25	1	69	3	1.00	1.000	0.11-29.70
RC	477	102	1981	358	0.844	0.180	0.66-1.07

[1]*P-value* obtained by Fisher's Exact test; **Abbreviations:** CT complexes of mtDNA in people with Parkinson's disease and controls: Control group; PD: People with Parkinson's disease; RC: Regulatory regions.
