# Supplementary material for: The mitogenome mutation repertoire affects progression of Parkinson’s Disease
Source: Genet Mol Biol. 2026 Feb 9;49(Suppl 4):e20250098. doi: 10.1590/1678-4685-GMB-2025-0098 (PMC12965417; doi:10.1590/1678-4685-GMB-2025-0098)
Supplement: Figure S4 [file 1415-4757-GMB-49-s4-e20250098-s6.pdf]

Genetics and Molecular Biology
https://doi.org/10.1590/1678-4685-GMB-2025-0098

Supplementary Material to "The mitogenome mutation repertoire affects progression of Parkinson's Disease"

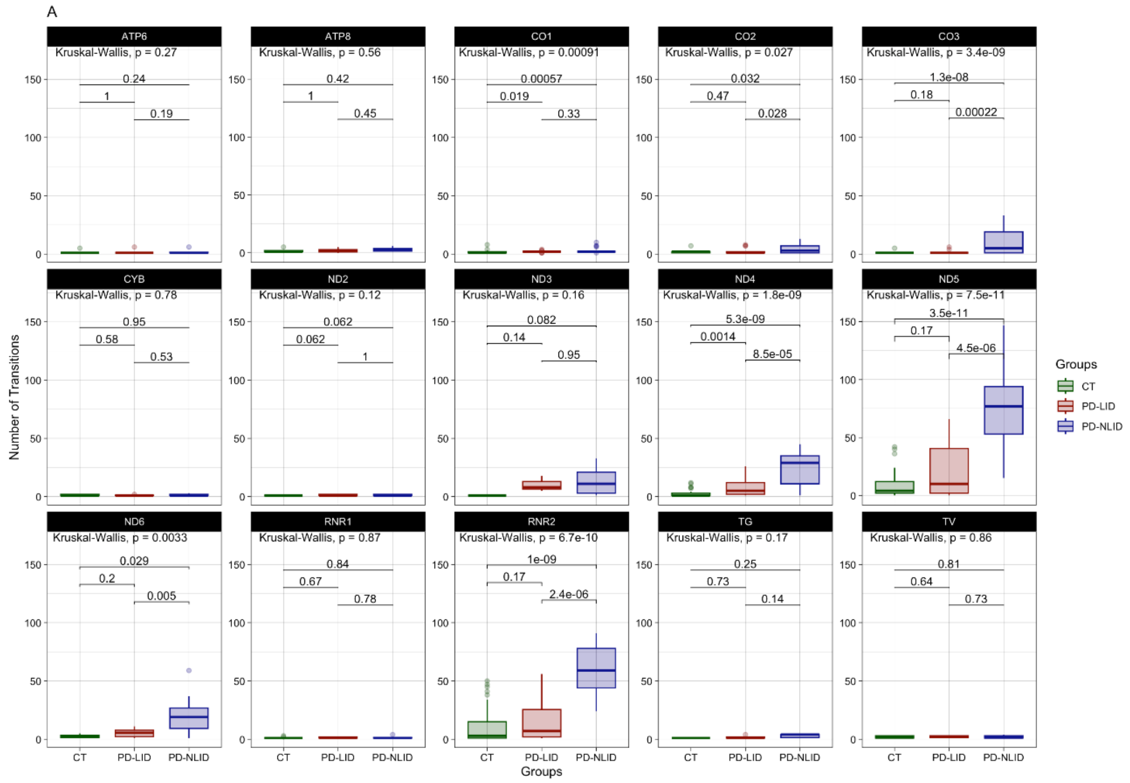

Figure S4 - Comparison of transition counts reveals statistical differences in seven mitochondrial genes (CO1, CO2, CO3, ND4, ND5, ND6, and RNR2) between the groups.
