# Supplementary material for: The mitogenome mutation repertoire affects progression of Parkinson’s Disease
Source: Genet Mol Biol. 2026 Feb 9;49(Suppl 4):e20250098. doi: 10.1590/1678-4685-GMB-2025-0098 (PMC12965417; doi:10.1590/1678-4685-GMB-2025-0098)
Supplement: Figure S5 [file 1415-4757-GMB-49-s4-e20250098-s7.pdf]

Genetics and Molecular Biology
https://doi.org/10.1590/1678-4685-GMB-2025-0098

Supplementary Material to "The mitogenome mutation repertoire affects progression of Parkinson's Disease"

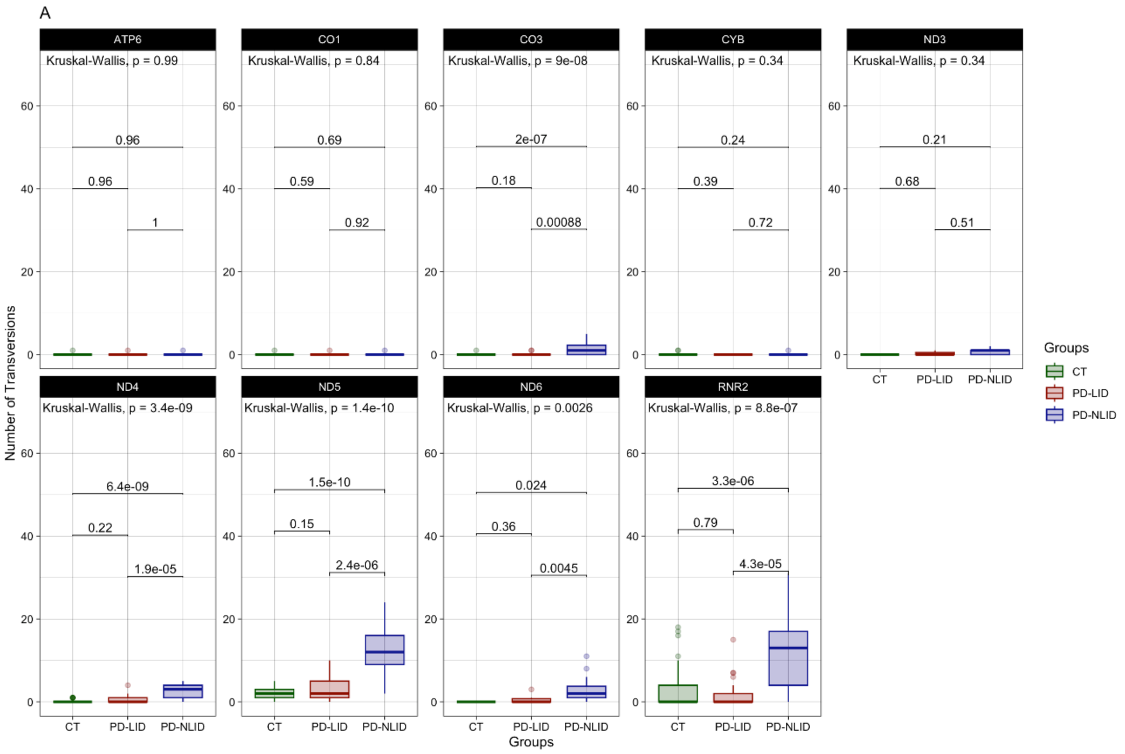

Figure S5 - Pairwise comparison of distribution of TVs highlights statistical differences in five mitochondrial genes (CO3, ND4, ND5, ND6 and MT-RNR2) across groups.
